# Supplementary material for: Persistent post-discharge opioid prescribing after traumatic brain injury requiring intensive care unit admission: A cross-sectional study with longitudinal outcome
Source: PLoS One. 2019 Nov 27;14(11):e0225787. doi: 10.1371/journal.pone.0225787 (PMC6880998; doi:10.1371/journal.pone.0225787)
Supplement: S1 Table — (DOCX) [file pone.0225787.s001.docx]

**S1 Table: Medication list**

| Non-steroidal Anti-Inflammatory Drugs (NSAIDs) | Aspirin |
| --- | --- |
|  | Ibuprofen |
|  | Naproxen |
|  | Aspirin-acetaminophen-caffeine |
|  | Diclofenac |
|  | Ketoprofen |
|  | Acetaminophen |
|  | Oxycodone-acetaminophen |
|  | Celecoxib |
|  | Hydrocodone-acetaminophen |
|  | Acetaminophen-codeine |
|  | Butalbital-acetaminophen |
|  | Meloxicam |
| Benzodiazepines/sedatives | Diazepam |
|  | Lorazepam |
|  | Alpraxolam |
|  | Clonazepam |
|  | Oxazepam |
|  | Midazolam |
|  | Trazodone |
|  | Zolpidem |
|  | Eszopicolone |
|  | Chlordiazepoxide |
| Antipsychotics | Aripriprazole |
|  | Quetiapine |
|  | Chlorpromazine |
|  | Clozapine |
|  | Haloperidol |
|  | Olanzapine |
|  | Risperidone |
| Antidepressants | Citalopram |
|  | Venlafaxine |
|  | Escitalopram |
|  | Amitriptyline |
|  | Nortriptyline |
|  | Sertraline |
|  | Desvenlafaxine |
|  | Fluoxetine |
|  | Paroxetine |
|  | Mirtazapine​ |
| Opioids | Fentanyl |
|  | Morphine |
|  | Hydromorphone |
|  | Oxycodone |
|  | Hydrocodone |
|  | Tramadol |
|  | Methadone |
|  | Remifentanil |
|  | Sufentanil |
|  | Oxycodone-acetaminophen |
|  | Hydrocodone-acetaminophen |
|  | Acetaminophen-codeine |
