# Supplementary material for: Persistent post-discharge opioid prescribing after traumatic brain injury requiring intensive care unit admission: A cross-sectional study with longitudinal outcome
Source: PLoS One. 2019 Nov 27;14(11):e0225787. doi: 10.1371/journal.pone.0225787 (PMC6880998; doi:10.1371/journal.pone.0225787)
Supplement: S2 Table — (DOCX) [file pone.0225787.s002.docx]

**S2 Table: Average total morphine equivalent dose administered per day and week between preadmission opioid users and opioid naïve patients.**

| **Exposure** | **Total n=431**  M ± SD [Min, Max] | **Preadmit opioid use n=76** M ± SD [Min, Max] | **Opioid naïve n=221**  M ± SD [Min, Max] | **p value** |
| --- | --- | --- | --- | --- |
| Sum ME (Day 1) | 1.41 ± 4.49 [0.00, 40.45] | 0.84 ± 1.79 [0.00, 13.60] | 1.18 ± 4.04 [0.00, 29.90] | 0.495 |
| Sum ME (Day 2) | 1.50 ± 6.33 [0.00, 74.57] | 0.75 ± 1.21 [0.00, 8.37] | 1.05 ± 3.77 [0.00, 34.74] | 0.539 |
| Sum ME (Day 3) | 1.17 ± 5.25 [0.00, 67.68] | 1.14 ± 3.09 [0.00, 19.92] | 1.00 ± 3.56 [0.00, 24.32] | 0.807 |
| Sum ME (Day 4) | 0.98 ± 5.16 [0.00, 66.19] | 0.80 ± 1.36 [0.00, 7.26] | 0.58 ± 1.66 [0.00, 12.56] | 0.472 |
| Sum ME (Day 5) | 0.94 ± 5.86 [0.00, 69.03] | 0.80 ± 1.53 [0.00, 6.98] | 0.29 ± 0.57 [0.00, 3.19] | **0.020** |
| Sum ME (Day 6) | 0.84 ± 5.29 [0.00, 53.25] | 0.43 ± 0.63 [0.00, 2.04] | 0.27 ± 0.56 [0.00, 2.21] | 0.336 |
| Sum ME (Day 7) | 0.30 ± 0.43 [0.00, 2.04] | 0.38 ± 0.52 [0.00, 2.04] | 0.25 ± 0.41 [0.00, 1.75] | 0.357 |
| Sum ME (Week 1) | 10.59 ± 37.78 [0.00, 370.23] | 5.60 ± 10.51[0.00, 62.19] | 5.47 ± 11.90 [0.00, 62.30] | 0.946 |
| Sum ME (Week 2) | 6.78 ± 17.94 [0.00, 130.92] | 4.80 ± 10.72 [0.00, 43.18] | 3.39 ± 6.83 [0.00, 33.03] | 0.56 |
| Sum ME (Week 3) | 3.30 ± 5.00 [0.00, 20.97] | 0.57 ± 0.61 [0.00, 1.56] | 5.86 ± 7.72 [0.00, 20.97] | 0.154 |
| Sum ME (Week 4) | 1.46 ± 1.96 [0.00, 8.60] | 0.78 ± 0.89 [0.12, 2.00] | 1.38 ± 1.31 [0.00, 3.35] | 0.442 |
| Sum ME (Week 5) | 0.77 ± 1.11 [0.00, 3.19] | 1.59 ± 2.26 [0.00, 3.19] | 0.33 ± 0.31 [0.00, 0.62] | 0.373 |
| Sum ME (Week 6) | 0.00 ± 0.00 [0.00, 0.00] | 0.00 ± 0.00 [0.00, 0.00] | 0.00 ± 0.00 [0.00, 0.00] | - |
| Sum ME (Week 7) | 1.25 ± 2.16 [0.00, 3.74] | 0.00 ± 0.00 [0.00, 0.00] | 0.00 ± 0.00 [0.00, 0.00] | - |
| Sum ME (Week 8) | 0.00 ± 0.00 [0.00, 0.00] | 0.00 ± 0.00 [0.00, 0.00] | 0.00 ± 0.00 [0.00, 0.00] | - |

Data presented as mean ± standard deviation [minimum, maximum].

Morphine equivalent (ME)
