# Supplementary material for: Persistent post-discharge opioid prescribing after traumatic brain injury requiring intensive care unit admission: A cross-sectional study with longitudinal outcome
Source: PLoS One. 2019 Nov 27;14(11):e0225787. doi: 10.1371/journal.pone.0225787 (PMC6880998; doi:10.1371/journal.pone.0225787)
Supplement: Attachment — Submitted filename: Manuscript Number-PONE-D-19-15116.doc [file pone.0225787.s004.doc]

Manuscript Number: PONE-D-19-15116

Article Type: Research Article

Full Title: Persistent Post-Discharge Opioid Prescribing after Traumatic Brain Injury Requiring

ICU Admission: A Cross-Sectional Study with Longitudinal Outcome

Short Title: Persistent Opioid Use After Traumatic Brain Injury

Corresponding Author: Lauren Kingsley Dunn

University of Virginia

Charlottesville, VA UNITED STATES

Keywords: traumatic brain injury; opioid dependence; opioid use disorder; Subarachnoid

hemorrhage; subdural hemorrhage

The authors have chosen to review a timely and relevant issue, opioid exposure and the sequelae. Their aim,

*“to determine incidence and risk factors for persistent opioid prescription after hospitalization for TBI.”*

The methodology employed was a retrospective review. The statistical analysis is noted to be appropriate.

There are however some limitations posed by the protocol design, the methodology and the conclusions, that must be noted/addressed:

#1. The premise of the study is an extrapolation of risk from the military TBI cohort. This is however difficult, as the TBI military cohort, also known as the Polytrauma cohort does not have a high specificity in identifying TBI. For many reasons, patients are included as TBI, allowing a high sensitivity in the diagnosis at the cost of specificity. These patients also have significant co-morbid injuries that impact comparisons of risk for opioid use. Thus any extrapolation is significantly limited.

#2. The exclusion of major injuries is noted,

*“to remove the confounding effect of non- neurological injuries on persistent post discharge opioid prescription…”*

However, there is no confirmation that these patients are isolated TBI. There are many other conflating injuries that might significantly impact pain and subsequent opioid use. These are not noted to be excluded. Further supporting this concern, is that spine surgical patients are included in the cohort.

#3. The TBI patients are not stratified by severity.

*“Occasionally high doses of opioids are employed for sustained periods of time in patients with a severe TBI requiring mechanical ventilation…”*

Severely injured patients are alluded to as needing sedation as a risk factor, but without stratification, it is unclear how the identified risk factors impacts the specific cohorts. As an example, mild patients would have less exposure and presumably less risk????

#4. The stratification of patients with pre versus post injury opioid use is of benefit. The findings however do not differ from the established understanding of risk associated with previous use. Unclear how TBI becomes an additional risk factor beyond previous use.

#5. The discussion section focuses on the perils of opioid use established, but fails to either clearly establish the risks from the study nor the link to the devastating sequelae highlighted.

In summary, it is agreed that the risk associated with opioid use in TBI patients is of concern. The manuscript however does not clearly identify risk factors specific to TBI beyond the known pre- injury use and further does not account for concomitant injuries.

Would recommend a cohort of isolated TBI versus those with additional injuries to better understand opioid use in TBI.

Would also recommend stratification of injury type, for better understanding of the risk assumed to exist in the severe cohort.
