# Supplementary material for: Persistent post-discharge opioid prescribing after traumatic brain injury requiring intensive care unit admission: A cross-sectional study with longitudinal outcome
Source: PLoS One. 2019 Nov 27;14(11):e0225787. doi: 10.1371/journal.pone.0225787 (PMC6880998; doi:10.1371/journal.pone.0225787)
Supplement: Attachment — Submitted filename: Response to Reviewers.docx [file pone.0225787.s006.docx]

**Response to Reviewers**

Reviewer #2: The authors have done a good job responding to most of my concerns. The table updates make the article easier to read. However, there is an outstanding issue:

My confusion regarding the modeling can be clarified if the introduction is changed. While I still do not think the current model answers their stated hypothesis (ICU opioid exposure as an independent variable affecting outcome) – it does answer a question. It appears that the authors are just searching for risk factors for persistent opioid prescription in patients with TBI. However, they couch all their data and discussion in terms of pre-admission opioid users and opioid naïve patients. The hypothesis in the introduction could be changed to the following:

“The aim of this study is to determine the incidence and risk factors for persistent opioid prescription (up to one year after admission) in patients with a primary TBI. We hypothesized that opioid use prior to hospitalization and in-hospital exposure to opioids for management of TBI would be associated with increased risk for persistent opioid prescription 1 year after hospital discharge.”
This would clarify why they present all the data stratified by pre-admission exposure vs naïve.

With this, it should be ready for publication from my standpoint.

We thank Reviewer 2 for their review of our revise manuscript. We have made the recommended change to the hypothesis stated in the Introduction Page 4, line 66-70.
